# Development and Evaluation of a Panel of Filovirus Sequence Capture Probes for Pathogen Detection by Next-Generation Sequencing

**DOI:** 10.1371/journal.pone.0107007

**Published:** 2014-09-10

**Authors:** Jeffrey W. Koehler, Adrienne T. Hall, P. Alexander Rolfe, Anna N. Honko, Gustavo F. Palacios, Joseph N. Fair, Jean-Jacques Muyembe, Prime Mulembekani, Randal J. Schoepp, Adeyemi Adesokan, Timothy D. Minogue

**Affiliations:** 1 Diagnostic Systems Division, United States Army Medical Research Institute of Infectious Diseases, Fort Detrick, Maryland, United States of America; 2 Pathogenica, Inc., Boston, Massachusetts, United States of America; 3 Virology Division, United States Army Medical Research Institute of Infectious Diseases, Fort Detrick, Maryland, United States of America; 4 Center for Genomic Sciences, United States Army Medical Research Institute of Infectious Diseases, Fort Detrick, Maryland, United States of America; 5 Metabiota, San Francisco, California, United States of America; 6 Institut National de Recherche Biomédicale, Kinshasa, Democratic Republic of the Congo; 7 Kinshasa School of Public Health, Kinshasa, Democratic Republic of the Congo; Division of Clinical Research, United States of America

## Abstract

A detailed understanding of the circulating pathogens in a particular geographic location aids in effectively utilizing targeted, rapid diagnostic assays, thus allowing for appropriate therapeutic and containment procedures. This is especially important in regions prevalent for highly pathogenic viruses co-circulating with other endemic pathogens such as the malaria parasite. The importance of biosurveillance is highlighted by the ongoing Ebola virus disease outbreak in West Africa. For example, a more comprehensive assessment of the regional pathogens could have identified the risk of a filovirus disease outbreak earlier and led to an improved diagnostic and response capacity in the region. In this context, being able to rapidly screen a single sample for multiple pathogens in a single tube reaction could improve both diagnostics as well as pathogen surveillance. Here, probes were designed to capture identifying filovirus sequence for the ebolaviruses Sudan, Ebola, Reston, Taï Forest, and Bundibugyo and the Marburg virus variants Musoke, Ci67, and Angola. These probes were combined into a single probe panel, and the captured filovirus sequence was successfully identified using the MiSeq next-generation sequencing platform. This panel was then used to identify the specific filovirus from nonhuman primates experimentally infected with Ebola virus as well as Bundibugyo virus in human sera samples from the Democratic Republic of the Congo, thus demonstrating the utility for pathogen detection using clinical samples. While not as sensitive and rapid as real-time PCR, this panel, along with incorporating additional sequence capture probe panels, could be used for broad pathogen screening and biosurveillance.

## Introduction

Filoviruses are highly pathogenic viruses that can cause outbreaks with significant disease and high lethality. Based on revised filovirus naming standards [1], the filoviruses are assigned to three different genera, *Ebolavirus*, *Marburgvirus*, and *Cuevavirus*. Five different ebolaviruses have been identified. They have at least 30% sequence divergence from virus to virus and include Ebola virus (EBOV), Sudan virus (SUDV), Taï Forest virus (TAFV), Reston virus (RESTV), and Bundibugyo virus (BDBV). Two individual viruses, Marburg virus (MARV) and Ravn virus (RAVV), are members of a single species of *Marburgvirus*.

The filovirus genome is nonsegmented, negative stranded RNA that contains seven genes encoding the nucleoprotein (NP), the viral proteins VP24, VP30, VP35, and VP40, the glycoprotein (GP), and the RNA-dependent RNA polymerase (L) protein. Recent outbreaks and newly discovered filoviruses have highlighted the geographic range and diversity of the filoviruses, including the identification of RESTV as a highly pathogenic virus of cynomolgus macaques [2] that leads to apparently asymptomatic infections in humans [3]. Additional filoviruses have recently been discovered, including BDBV in Uganda [4], and Lloviu virus (LLOV), the only member of the genus *Cuevavirus*, in Spain [5].

In regions of the world endemic for the malaria parasite, infection with highly pathogenic viruses can be misdiagnosed, leading to delays in appropriate treatment and improper patient isolation. This misdiagnosis can result in an incomplete understanding of the pathogens circulating in a particular region, impacting preparation for an outbreak response. A recently published study found that most of the acute patients admitted to the Lassa Fever Ward in Sierra Leone, a region hyperendemic for Lassa fever and malaria, were Lassa fever virus (LASV) antigen negative [6]. Of these LASV-negative patients, approximately 25% were IgM positive for Rift Valley fever virus, West Nile virus, Chikungunya virus, or the filoviruses EBOV and MARV. Supporting this study's findings and highlighting the need for increased, broad pathogen biosurveillance is the current widespread outbreak of Ebola virus disease in West Africa, including Sierra Leone [7].

Real-time PCR and pathogen-specific ELISAs are commonly used for pathogen detection and biosurveillance studies. While these assays, especially real-time PCR, are highly sensitive and specific, screening multiple samples for an unknown pathogen can be expensive, time consuming, and limited for broad pathogen screening studies due to small sample volume. One solution to this is the use of multiplexed real-time PCR assays [8]–[12], allowing multiple targets to be assessed in a single sample. However, limitations to the number of assays capable of being multiplexed, the number of discrete dyes for separating multiplexed results, and the inherent specificity for the targeted agents leave this technique relatively undeveloped for broad pathogen screening. One alternative strategy for screening for a large number of pathogens is the use of next-generation sequencing (NGS), a maturing tool for the identification of known and unknown pathogens from both clinical and environmental samples [13], [14]. While directly sequencing DNA or RNA within a sample using NGS can be advantageous, significant amounts of the sequencing data generated are almost exclusively host or non-target metagenomic sequences as opposed to pathogen sequence. This results in a large bioinformatic burden for discriminating pathogen sequence from background in addition to the added cost of sequencing the background DNA or RNA. Additionally, the lack of specificity, which is a great strength to the NGS approach, is a significant hurdle for progressing any diagnostic through FDA clearance.

**Figure 1:**
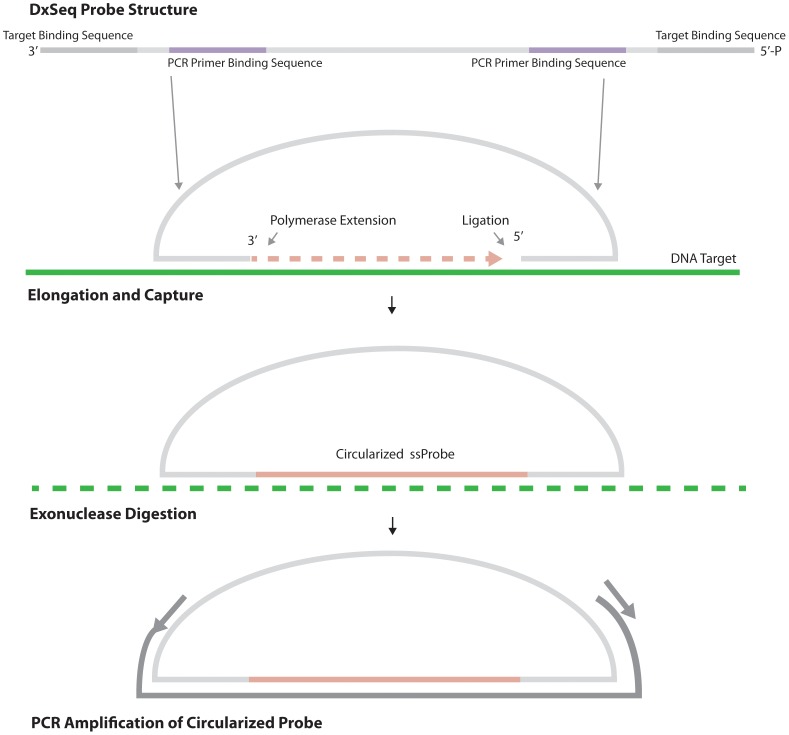
Overview of the DxSeq technology. Linear oligonucleotide probes contain complementary sequences that hybridize to the targeted sequence. A polymerase fills in the target sequence, and a ligation reaction captures the sequence within the circularized probe (Circularized ssProbe). Exonucleases remove noncircularized probe and DNA within the reaction, and the captured sequence is amplified by PCR using primers within the probe that bridge the captured sequence.

One approach to minimize unwanted sequencing and add specificity to the assay is the use of specific sequence capture probes (SCPs) followed by identification by NGS (DxSeq technology, Figure 1). These probes contain a conserved probe backbone comprised of primer binding sequences, allowing amplification of the captured sequence by PCR, and a short linker sequence between the primer binding sequences. The probe has 3′ and 5′ ends that are complementary to the targeted sequence; when the probe is added, these ends hybridize to the target DNA, flanking the sequence to be captured within the probe. An enzymatic polymerization and ligation fill-in reaction captures the sequence within the probe, and a subsequent PCR bridging the captured sequence amplifies the target for sequencing. Multiple probes having similar hybridization temperatures can be combined into a single probe mixture, allowing for the capture and identification of hundreds to thousands of targets [15], [16], and multiple samples can be run in a single sequencing reaction using sample-identifying indices. This approach combines multiple layers of specificity including two probe arm hybridization events, PCR amplification using two different primers, and ultimately captured sequence identification by NGS.

In this context, we developed a panel of pathogen-specific probes to detect multiple filoviruses including EBOV, SUDV, RESTV, TAFV, BDBV, and the MARV variants Musoke, Ci67, and Angola. Design and evaluation of these multiple probes led to a final pooled panel of 48 filovirus probes that successfully identified cell culture amplified virus and the etiologic agent from nonhuman primate (NHP) plasma samples and human sera samples.

## Methods

### Ethics statement

The samples from rhesus macaques used in this study were archived and were not collected for the purpose of this study. The NHP experiment and procedures were approved by the USAMRIID Institutional Animal Care and Use Committee (IACUC) and was carried out in compliance with the regulations outlined in the USDA Animal Welfare Act (PHS Policy) and other Federal statutes and regulations relating to animals and experiments involving animals. The facility where this research was conducted is accredited by the Association for Assessment and Accreditation of Laboratory Animal Care, International and all animal work done adhere to the conditions specified in the Guide for the Care and Use of Laboratory Animals (National Research Council, 2011). Animals were given enrichment (including toys and mirrors) regularly as recommended by the Guide for the Care and Use of Laboratory Animals. Food was provided (commercial biscuits, fruit), and animals were checked at least daily according to the protocol. All efforts were made to minimize painful procedures; the attending veterinarian was consulted regarding painful procedures, and animals were anesthetized prior to phlebotomy. Following the development of clinical signs, animals were checked multiple times daily. When clinical observations and scores of animals reached defined levels based on the approved IACUC protocol (scores based on a combination of responsiveness, recumbency, and clinical signs), animals were euthanized by exsanguination following deep anesthesia and administration of a pentobarbital-based euthanasia solution to minimize pain and distress. All animals were housed at USAMRIID.

De-identified human sera samples from individuals infected with BDBV in the Democratic Republic of the Congo (DRC) were used in this study. These samples were determined by the institutional Office of Human Use and Ethics to be Not Human Subject Research (HP-12-15). All samples were collected and de-identified in the DRC. The samples had indirect identifiers when we received them that prevented us from matching samples to the data that was collected. None of the samples were collected for this study; this study was an additional use of the samples collected for another purpose. Informed and written consent was obtained, and author JJM was involved in the collection of the samples.

### Viral RNA

Viruses used in this study include the ebolaviruses SUDV, EBOV, RESTV, TAFV, and BDBV. MARV variants included Musoke, Ci67, and Angola. All viruses are maintained at USAMRIID, and IRB approval was not required for use. For each of the filoviruses, total RNA was purified from the supernatant of virus-infected cells using TRIzol (Life Technologies, Grand Isle, NY). Total nucleic acid from 100 µl cell culture supernatant was isolated directly from the TRIzol mixture using the EZ1 Virus 2.0 kit (Qiagen, Valencia, CA) and the EZ1 robot (Qiagen) according to the manufacturer's directions. Nucleic acid was eluted in a final volume of 60 µl elution buffer.

### Plasma and sera samples

Archived NHP plasma samples were used in this study. Animals were challenged by the intramuscular route with approximately 300 plaque forming units (PFU) of EBOV H. sapiens-tc/COD/1995/Kikwit, and longitudinal samples were taken over the course of the infection. Whole blood collected into K2EDTA tubes or serum clot activator tubes with gel separator (Vacuette, Greiner Bio-One, Monroe, NC) was centrifuged at room temperature to separate plasma/sera in accordance with manufacturer's guidance. RNA samples were prepared using a 1 part sample: 3 parts reagent ratio into TRIreagent LS (Sigma Aldrich, St. Louis, MO). Aliquots were prepared and stored at −80°C until analysis. Serum viremia for NHP samples were titrated by plaque assay using Vero E6, a 1% SeaKem agarose (Lonza, Allendale, NJ) primary overlay, and a 5% neutral red secondary overlay. Log dilutions of sera were prepared and plated in replicates of three to six, and a tissue-culture EBOV seed was included in half-log dilutions as a positive control. Human serum samples from individuals infected with BDBV were also used in this study. Reference materials, including aliquots of diagnostic samples, were obtained through a collaboration with National Institute of Biomedical Research (French acronym INRB) in Kinshasa. Virus from potentially positive samples was amplified by infecting a monolayer of Vero E6 cells and looking for cytopathic effects (CPE).

Total nucleic acid from 17.5 µl human serum samples and 17.5 µl of cell culture supernatant from cell cultures with CPE was isolated by TRIzol extraction (elution volume of 70 µl AE buffer) using the QIAamp Viral RNA Mini Kit (Qiagen) according to the manufacturer's directions. For the NHP samples, 25 µl plasma was extracted with TRIzol and eluted in 100 µl AE buffer. Presence of virus for the human samples was determined using established real-time PCR assays for the SUDV, EBOV, RESTV, TAFV, BDBV, RAVV,and the MARV variants Musoke, Ci67, and Angola, and the presence of EBOV was determined in the NHP samples as previously described [17]. Due to the available sample volume, samples were run in duplicate (human) or in singlet (NHP) using 5 µl of purified RNA on the LightCycler 2.0 (Roche Diagnostics Corporation, Indianapolis, IN). A positive sample was defined as having a C_Q_ value of <40 cycles.

### DxSeq probe design

Reference sequences for the filoviruses SUDV (GenBank # NC_006432), EBOV (GenBank # NC_002549), RESTV (GenBank # NC_004161), TAFV (GenBank # NC_014372), BDBV (GenBank # NC_014373), and MARV variants Musoke (GenBank # NC_001608), Ci67 (GenBank # EF446132), and Angola (GenBank # NC_DQ447660.1) were analyzed to identify conserved regions for probe hybridization that would also contain viral sequence that would differentiate the filovirus isolates during sequence analysis. Pathogenica's probe design algorithms can analyze hundreds or thousands of genomes simultaneously to determine which regions to target to maximize the information contained in the sequences. In addition, the probe design algorithm allows for the production of a multiplex set of non-interacting probes. These are specifically selected to provide discrimination between closely related viruses that differ by 1 or more bases at multiple loci within the viral genome. Based on the *in silico* analyses, panels of 15 probes for each filovirus were designed to capture approximately 100 bases of viral sequence. Probes were synthesized by Integrated DNA Technologies (Coralville, IA), and full sequences are available from Pathogenica, Inc, upon request. Please see Table S1 for the probe arm binding sites and the filovirus specific capture sequence for all of the probes included in the final filovirus probe panel.

### Sample processing

For the initial probe evaluation, total RNA from the cell culture supernatant of cells infected with stock virus was amplified from 50 ng total nucleic acid using the Quantitect Whole Transcriptome Amplification Kit (Qiagen) according the manufacturer's protocol. Probe was hybridized to 50 ng amplified cDNA by adding 3 nM probe in 1.5x ampligase buffer (Epicentre, Madison, WI) and heating the reaction to 94°C for 2 minutes and then to 60°C (0.1°C/sec temperature decrease) with a 60 min hold at 60°C. Target sequence was captured within the probe by adding 2 units AmpliTaq Stoffel Fragment (Life Technologies) and 4 units Ampligase (Epicentre) to the reaction and incubating at 60°C for 60 min with a final concentration of 0.4 µM dNTPs (BioFire, Salt Lake City, UT).

Noncircular probes and background DNA were removed by heating the reaction to 94°C for 2 min, ramping to 37°C, and adding 10 units Exonuclease I (New England Biolabs, Ipswich, MA) and 50 units exonuclease III (New England Biolabs) to the reaction. Samples were incubated at 37°C for 30 min followed by enzyme inactivation at 94°C for 15 min. Captured sequence was amplified by Platinum *taq* (Life Technologies) using probe-specific primers designed to amplify the captured region [0.4 µM forward (5′-CAGATGTTATCGAGGTCCGAC) and reverse (5′-GGAACGATGAGCCTCCAAC) primers]. Reactions were amplified at 95°C for 3 min, 40 cycles of 95°C for 30 sec, 60°C for 30 sec, 72°C for 1 min, and a final hold at 72°C for 10 min on the MJ Mini (Bio-Rad, Hercules, CA) or T100 thermocycler (Bio-Rad). Following the PCR amplification of the captured sequences, amplicons were resolved on a 2% agarose gel and purified using the QIAQuick Gel Extraction Kit (Qiagen). Expected amplicon sizes were from 180 to 200 base pairs. Samples were quantified using the Bioanalyzer (Agilent Technologies, Santa Clara, CA) with the DNA 1000 kit.

All of the filovirus probes were initially evaluated using amplified, strain specific filovirus cDNA. Individual probes that yielded a PCR product of the appropriate size were pooled into the final filovirus probe panel. This panel was further evaluated with each of the amplified filovirus cDNAs using the protocol described above.

### Sequencing and analysis

Gel-purified amplicons were sequenced on the MiSeq or the GAIIx platform (Illumina, San Diego, CA) using the 2×150 cycle sequencing kit. Libraries were prepared using the TruSeq DNA Sample Preparation Kit (Illumina) with single (NHP samples) or dual (human samples) indices. The resulting sequencing reads were analyzed using CLC Genomics Workbench (CLC Bio, Cambridge, MA). Reads were trimmed for quality, and the primer sequences used for the capture sequence amplification were trimmed. Reads were further trimmed based on length, removing reads less than 100 bases. The trimmed and filtered reads were then mapped to a reference library containing the targeted capture sequences and each filovirus genome (GenBank NC_002549.1, NC_004161.1, NC_006432.1, NC_014373.1, EF446132.1, NC_001608.3, NC_014372.1, and DQ447660). Mapping settings included 70% of the read had to match the reference sequence by at least 80% identity, and nonspecific read mappings were ignored. Reads that mapped to the respective filovirus strain was normalized by determining the percentage of the total sequencing reads following trimming and filtering that successfully mapped. For the human samples, 5 no template controls (NTCs) were included, and a sample was called positive if the percentage of mapped reads or the total number of reads was greater than the NTC average plus three times the standard deviation of the NTCs.

## Results

### Sequence capture probe identification

Multiplex SCP assays can target divergent genomic signatures for strain identification and differentiation by capturing a conserved signature within a probe followed by sequencing. The 3′ and 5′ probe arms contain sequences that are complementary to the viral sequence flanking the filovirus-specific target sequence, allowing the conserved sequence to be captured for identification by sequencing. To identify these filovirus-specific target signatures, the filovirus genomes for SUDV, EBOV, RESTV, TAFV, BDBV, and the MARV variants Musoke, Ci67, and Angola were aligned. Multiple sequences within each viral genome were identified for probe design, each having a conserved, filovirus-specific sequence. This conserved sequence had to be flanked by additional sequence such that the probe's complementary arms would hybridize at >60°C. This analysis resulted in a panel of 15 sequence capture probes per viral strain.

### Filovirus DxSeq probe analysis

An upfront amplification protocol prior to sequence capture was developed and optimized in order to maximize sensitivity from limited sample volumes. Initially, using five of the SUDV DxSeq probes, a comparison of cDNA generation using Superscript II (Life Technologies) and Qiagen's Whole Transcriptome Amplification (WTA) kit resulted in increased sensitivity, measured by amplicon signal, using the WTA kit (data not shown). Using these same probes, optimization of cDNA concentration showed 50 ng cDNA had increased amplicon yield (data not shown). Testing of all of the filovirus probes individually for generation of appropriately sized amplicon under these conditions allowed for a downselection to the final filovirus DxSeq panel development. Specific information regarding location of the probes within this final panel, including the capture sequence locations, can be found Table S1.

Evaluation of the pooled filovirus DxSeq panel with cell culture supernatant RNA from virus-infected cells resulted in positive detection of relevant virus with limited cross-reactivity with other filovirus variants (Table 1). A representative mapping for SUDV is shown in Figure S1. In these analyses cDNA samples were derived from virus infected cell culture supernatant that ranged from 4×10^5^ to 1.1×10^7^ pfu/ml. Overall, the pooled probe panel showed very high specificity for the respective filovirus with little cross-reactivity among the different variants (Table 1). For most of these high-titer samples, the percentage of total reads that mapped to the target filovirus genome ranged from 5–91%. Surprisingly, the probes designed to detect RESTV did not generate RESTV-specific sequence when used in the probe pool even though these probes successfully captured the target sequence when used individually. Since these probes did individually detected RESTV by sequencing, these 3 probes were still included in the final probe panel.

**Table 1:** Filoviruses differentiation using a pooled sequence capture probe panel.

		Reference sequence															
		Ebola		Sudan		Taï Forest		Bundibugyo		Reston		Ci67		Musoke		Angola	
RNA	Reads^1^	Mapped	%^2^	Mapped	%^2^	Mapped	%^2^	Mapped	%^2^	Mapped	%^2^	Mapped	%^2^	Mapped	%^2^	Mapped	%^2^
Ebola	570,783	475,687	83.34	11,993	2.10	225	0.04	151	0.03	0	0.00	16	0.00	0	0.00	29	0.01
Sudan	631,885	3,668	0.58	569,003	90.05	12	0.00	118	0.02	0	0.00	26	0.00	0	0.00	34	0.01
Taï Forest	376,987	26	0.01	37	0.01	193,387	51.30	193	0.05	0	0.00	14	0.00	0	0.00	45	0.01
Bundibugyo	705,687	26	0.00	57	0.01	37	0.01	642,273	91.01	0	0.00	56	0.01	30	0.00	71	0.01
Reston	141,540	40	0.03	48	0.03	112	0.08	307	0.22	0	0.00	48	0.03	0	0.00	21	0.01
Ci67	482,949	38	0.01	31	0.01	23	0.00	196	0.04	0	0.00	217,446	45.02	482	0.10	190	0.04
Musoke	279,685	332	0.12	58	0.02	107	0.04	1510	0.54	0	0.00	50	0.02	15,171	5.42	88	0.03
Angola	429,471	24	0.01	37	0.01	28	0.01	178	0.04	0	0.00	159	0.04	448	0.10	259,714	60.47

1 Reads are the total number of reads after trimming and filtering;

2 % refers to the percentage of the total reads after trimming and filtering that mapped to the respective reference sequence.

### Probe evaluation using clinical samples

**Figure 2:**
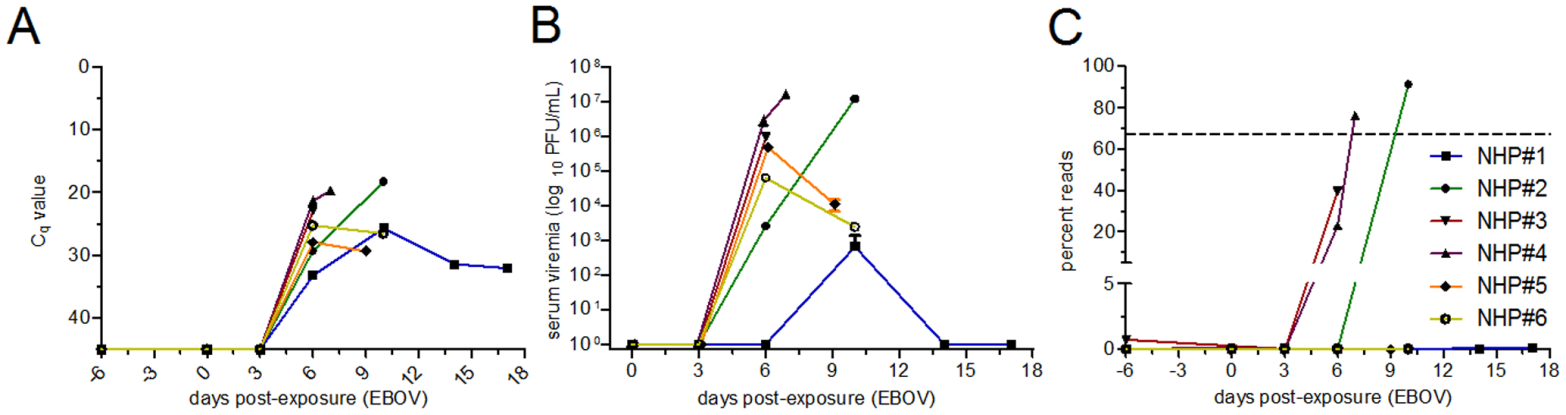
Detection of EBOV in challenged NHPs. Plasma samples from each NHP were assayed for the presence of EBOV by real-time PCR (A) and by plaque assay (B). Samples were run singly due to limited plasma availability. (C) Plasma samples were also assessed using the filovirus SCP panel. The dashed line indicates the signal generated by the EBOV positive control.

Archived plasma samples from NHPs experimentally challenged with EBOV were acquired for this study. Relative amounts of virus from RNA extracted from these samples were determined by real-time PCR (Figure 2A), and serum viremia was quantified by plaque assay titration (Figure 2B). RNA from these samples was also screened using the filovirus DxSeq SCP panel, successfully identifying EBOV in three of the six challenged NHPs (Figure 2C) with minimal reads mapping to the other filovirus genomes (see Table S2 for read mapping against each filovirus genome). Real-time PCR showed higher sensitivity than SCP detection; however, the filovirus panel correctly identified EBOV in the three animals having the highest viral load. Of note, we did observe read misidentification due to read index demultiplexing. For example, reads that mapped to EBOV were found in samples that should be negative and were negative by real-time PCR (ex. the pre-challenge day -6 sample from NHP#3). To more clearly determine the positive/negative cutoff, five NTCs were included in the subsequent sequencing reactions, and a positive was defined as being greater than the NTC average plus 3 times the standard deviation.

A series of de-identified human sera samples from patients infected with BDBV from the DRC [18] were subsequently assessed using the filovirus probe panel. These samples were from individuals that had a potential BDBV exposure (some clinically negative at the time of sample collection), but all individuals did eventually show clinical signs of infection. Real-time PCR of these sera samples and the cell culture supernatant from cell culture based viral amplification identified all of the supernatants and half of the sera samples as being positive for BDBV (Table 2). Real-time PCR testing of the whole transcriptome amplified RNA from these samples found all but one of the positive sera samples positive. Of note, though, is a decrease in the real-time PCR signal following the WTA reaction that likely resulted in non-detection using samples near the clinical limit of detection (sample 2012-176, Table 2).

**Table 2:** Real-time PCR identification of BDBV in human clinical sera samples.

		Sample		Amplified sample	
Sample^1^	Matrix	C_T_ 2	P/N	C_T_	P/N
2012-1	serum	21.96/21.75	P	31.42/30.45/29.69	P
	cell supernatant-1	14.73/14.76	P	18.31/18.27/17.97	P
	cell supernatant-2	15.20/15.52	P	19.66/19.61/19.66	P
2012-16	serum	45/45	N	45/45/45	N
2012-91	serum	45/45	N	45/45/45	N
2012-95	serum	45/45	N	45/45/45	N
2012-99	serum	45/45	N	45/45/45	N
2012-120	serum	21.07/21.02	P	30.89/30.82/31.36	P
	cell supernatant	15.20/15.21	P	19.23/19.23/19.29	P
2012-147	serum	22.85/22.90	P	32.24/32.69/32.97	P
	cell supernatant	14.91/14.95	P	18.45/18.45/18.45	P
2012-153	serum	25.08/24.92	P	35.85/33.74/34.91	P
	cell supernatant	15.24/14.95	P	18.71/18.74/17.50	P
2012-176	serum	32.60/32.18	P	45/45/45	N
2012-198	serum	45/45	N	45/45/45	N
2012-014	cell supernatant	15.56/15.39	P	20.36/19.99/20.19	P

1 For samples not having a corresponding cell supernatant sample, no CPE was observed during viral amplification;

2 Sera samples were run in duplicate due to limited sample availability.

Using the filovirus SCPs panel, all of the cell culture amplified virus, which were all positive by real-time PCR, had positive identification of BDBV in the samples (Table 3, run 1). During the first sequencing run, all of the sera samples were negative using the filovirus SCPs panel, likely due to a high background among the NTCs. To improve positive/negative calling, we re-sequenced these libraries, removing the cell culture amplified samples and decreasing the amount of the positive control library used in the sequencing run. This resulted in decreased read misidentification and lower background as noted in the BDBV reads among the NTC samples (Table 3, run 2). Three of the five BDBV real-time PCR positive samples were also positive using the filovirus SCPs panel, one real-time PCR presumptive negative returned positive results for BDBV, and two sera samples (one of the real-time PCR positive samples and one of the negative samples) had mixed results (positive using the read cutoff but negative using the percentage of mapped reads cutoff). Similar to the NHP analyses, read mapping of the human samples showed minimal mapping against the other filovirus genomes (Tables S3 and S4).

**Table 3:** Screening human clinical sera samples identifies BDBV.

		Filovirus probe panel run 1					Filovirus probe panel run 2				
Sample^1^	Matrix	Reads	Mapped	P/N	%	P/N	Reads	Mapped	P/N	%	P/N
2012-1	serum	145,553	845	N	0.581	N	208,322	3200.154	N	0.154	N
	supernatant-1	638,437	568,626	P	89.065	P	Ns				
	supernatant-2	840,456	569,439	P	67.754	P	Ns				
2012-16	serum	828,309	1,997	N	0.241	N	1,132,782	825	P	0.073	N
2012-91	serum	255,862	431	N	0.168	N	344,943	283	N	0.082	N
2012-95	serum	248,909	811	N	0.326	N	343,551	364	N	0.106	N
2012-99	serum	201,613	1,223	N	0.607	N	280,256	965	P	0.344	P
2012-120	serum	211,015	1,309	N	0.620	N	279,390	800	P	0.286	P
	supernatant	604,371	527,607	P	87.299	P	Ns				
2012-147	serum	183,639	2,070	N	1.127	N	243,107	870	P	0.358	P
	supernatant	967,611	852,653	P	88.119	P	Ns				
2012-153	serum	177,170	1,303	N	0.735	N	227,555	947	P	0.416	P
	supernatant	709,431	565,642	P	79.732	P	Ns				
2012-176	serum	324,518	842	N	0.259	N	447,698	294	N	0.066	N
2012-198	serum	321,721	1,182	N	0.367	N	447,698	676	P	0.151	N
	supernatant	802,109	713,666	P	88.974	P	Ns				
Positive		727,421	684,988	P	94.167	P	119,617	102,060	P	85.322	P
NTC1		153,857	934	N	0.607	N	292,952	369	N	0.126	N
NTC2		170,488	572	N	0.336	N	232,024	16	N	0.007	N
NTC3		311,773	324	N	0.104	N	430,436	13	N	0.003	N
NTC4		307,239	806	N	0.262	N	412,609	40	N	0.010	N
NTC5		133,303	1,462	N	1.097	N	148,100	21	N	0.014	N
		Cutoff^2^	2,104		1.649		Cutoff^2^	558		0.190	

1 For samples not having a corresponding cell supernatant sample, no CPE was observed during viral amplification;

2 Cutoff is equal to the average of the no template controls (NTC) plus 3 times the NTC standard deviation; P  =  positive call, N  =  negative call, ns  =  not sequenced.

## Discussion

A critical factor for deciding on a diagnostic assay panel for patient testing is the diversity of the pathogens circulating in a particular geographic region, and incomplete biosurveillance data can lead to inappropriate diagnostics and capacity building. For example, a recent study showed most (60–70%) of the samples from the Lassa Fever Ward in Sierra Leone, a region endemic for Lassa fever and malaria, were negative for both of these pathogens. Some of these negative samples showed evidence of an acute infection with, among other viruses, the filoviruses EBOV and MARV [6]. This study was published during the current Ebola virus disease outbreak in West Africa, but increased biosurveillance could have impacted pathogen screening and response plans for a filovirus disease outbreak in this geographic region.

Having the capability to screen a clinical or environmental sample for multiple viruses or bacteria of interest within a single reaction would be a significant improvement for pathogen identification and biosurveillance. Screening a sample for multiple targets can become expensive when testing for a single target at a time, and large scale screening for multiple pathogens could be limited by a small sample volume. Multiplexing assays can address these concerns, and many multiplexed real-time PCR assays have been developed. For example, one assay can identify four different dengue virus serotypes [8], and another can differentiate *Burkholderia pseudomallei* from *B. mallei* [12].

NGS is another technology that can theoretically identify any pathogen in a sample, but this lack of specificity can be a hurdle for getting FDA approval for a diagnostic. Targeted sequencing gives specificity to NGS, allowing many targets to be assayed simultaneously in a single tube reaction. Commercially available panels with >400 probes are available, and the underlying technology has been demonstrated capable of assaying >10,000 loci simultaneously [16]. In this study, SCPs were developed and evaluated as a multiplexed NGS assay capable of detecting and differentiating multiple filoviruses with a high degree of specificity. Coupled to NGS, each of the probe sets was highly specific, generally having little cross-reactivity with the other related filoviruses.

Testing of human and NHP clinical samples using this panel resulted in positive detection of BDBV and EBOV, respectively. While not as sensitive as real-time PCR, the filovirus SCP panel correctly identified EBOV in the three experimentally infected NHPs having the highest viral loads, and BDBV was correctly identified in multiple human clinical sera and cell culture amplified samples. In terms of assay sensitivity, this panel correctly identified the targeted virus at high concentrations, but there was a loss of detection at the samples containing the lower amounts of virus as determined by real-time PCR. This lower sensitivity near the limit of detection likely resulted from the inherent nature of NGS analysis and barcode demultiplexing, reflected in higher background signals among the negative and NTC samples. A previous study found an ∼0.3% read misidentification rate for single indexed samples [19]. For the human clinical samples, this read misidentification impact was minimized by decreasing the amount of the high target samples and resequencing. Having appropriate confirmatory assays, such as real-time PCR or antigen capture, would still be required for sample detection near the limit of detection. An additional reason for the lower level of detection was a dilution of the viral sequence following the whole transcriptome amplification. Future studies would be needed to identify alternatives to this amplification step in order to improve detection of low abundance targets and increase the time-to-answer.

Overall, we developed an expandable, broad filovirus detection panel that could aid in biosurveillance and diagnostics. While not as sensitive or rapid as real-time PCR, there is added benefit of being able to screen multiple samples for a large number of pathogens using a small sample volume. Regional-specific, syndromic, or biothreat detection panels could be developed by incorporating additional SCPs, ultimately leading to a broad pathogen detection system for use in biosurveillance.

## Supporting Information

Figure S1 **Read mapping to the SUDV genome shows specific reads mapping to the targeted capture sequence regions.** The combined filovirus probe panel was evaluated using RNA from multiple filoviruses. Shown is the mapping of the reads against the SUDV genome using SUDV RNA is the starting material. There were 569,003 reads (90.05%) of 631,885 post-trimming and filtering reads that mapped to the SUDV reference genome.
(TIF)

Table S1 **Filovirus probe hybridization and capture sequence.**
(DOCX)

Table S2 **Detailed read mapping for the nonhuman primate clinical sera samples using the filovirus probe panel.**
(DOCX)

Table S3 **Detailed read mapping for the human clinical sera samples using the filovirus probe panel (run 1).**
(DOCX)

Table S4 **Detailed read mapping for the human clinical sera samples using the filovirus probe panel (run 2).**
(DOCX)
