# Supplementary figures and images for: Development and Evaluation of a Panel of Filovirus Sequence Capture Probes for Pathogen Detection by Next-Generation Sequencing
Source: PLoS One. 2014 Sep 10;9(9):e107007. doi: 10.1371/journal.pone.0107007 (PMC4160210; doi:10.1371/journal.pone.0107007)

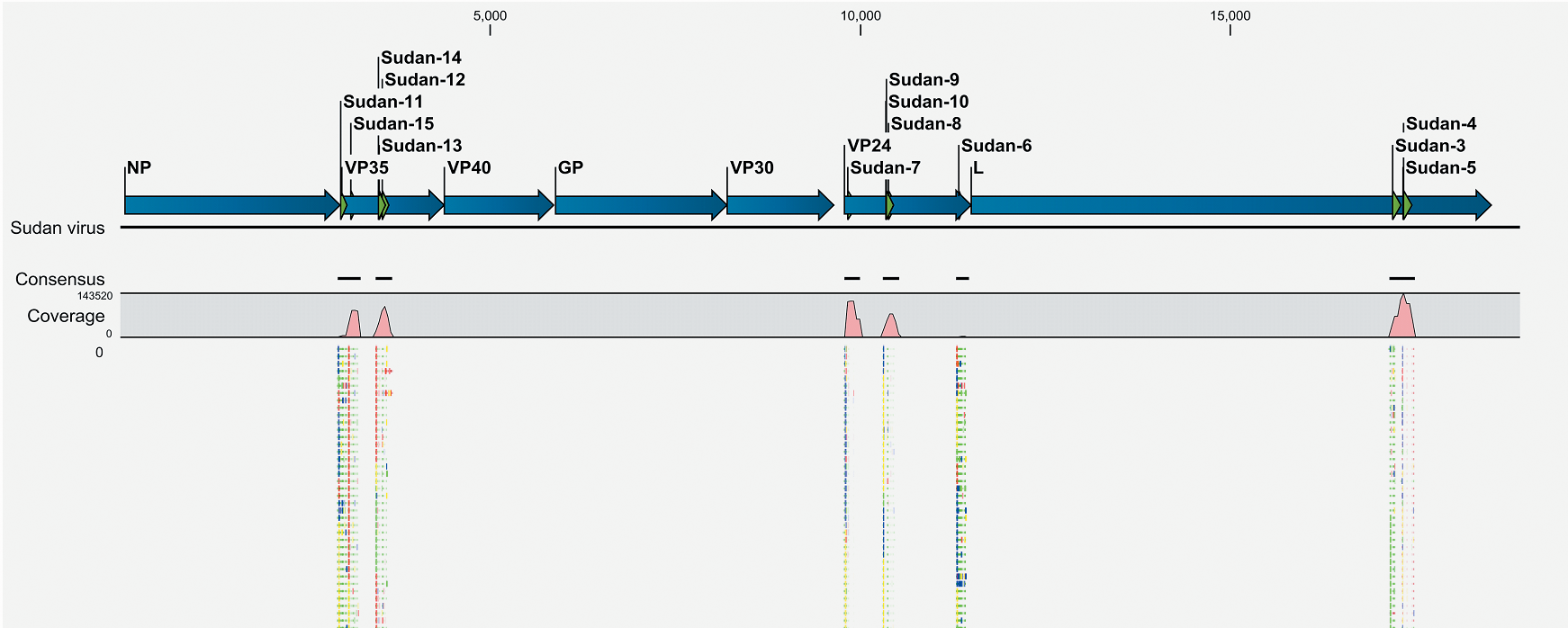

Supplement: Figure S1 — Read mapping to the SUDV genome shows specific reads mapping to the targeted capture sequence regions. The combined filovirus probe panel was evaluated using RNA from multiple filoviruses. Shown is the mapping of the reads against the SUDV genome using SUDV RNA is the starting material. There were 569,003 reads (90.05%) of 631,885 post-trimming and filtering reads that mapped to the SUDV reference genome. (TIF) [file pone.0107007.s001.tif]
