# Supplementary material for: Development and Evaluation of a Panel of Filovirus Sequence Capture Probes for Pathogen Detection by Next-Generation Sequencing
Source: PLoS One. 2014 Sep 10;9(9):e107007. doi: 10.1371/journal.pone.0107007 (PMC4160210; doi:10.1371/journal.pone.0107007)
Supplement: Table S1 — Filovirus probe hybridization and capture sequence. (DOCX) [file pone.0107007.s002.docx]

**Table S1: filovirus probe hybridization and capture sequence.**

| **probe** | **location** | **gene** | **sequence (5’-3’)^1^** |
| --- | --- | --- | --- |
| Bundibugyo-1 | 18,346-18,497 | L | GCACATGCAGGTTCCTGTTGAGCTTTAAAAGATCATGCAATATAAAATGATATTTGTATACTAATCATGTTAGTACTAACTAACAGTACTCACTGCATATACTCTATCAATTAAGAAAAATTACTGTGGTTTATGCATTTAAATGACATCAC |
| Bundibugyo-2 | 18,346-18,498 | L | GCACATGCAGGTTCCTGTTGAGCTTTAAAAGATCATGCAATATAAAATGATATTTGTATACTAATCATGTTAGTACTAACTAACAGTACTCACTGCATATACTCTATCAATTAAGAAAAATTACTGTGGTTTATGCATTTAAATGACATCACA |
| Bundibugyo-3 | 13,425-13,576 | L | AGAATGCCACTGTTAGAGGCAGCAGTTTTGTTACCGACCTAGAAAAATACAACTTGGCATTTAGATATGAGTTTACAGCTCCATTTATTGAATACTGTAATCGATGTTATGGTGTAAAAAATTTATTCAATTGGATGCATTATACGATACCG |
| Bundibugyo-4 | 11,837-11,988 | L | CCTGTCGAACCAAAGTGCAGCCAATTCTTAGATGAAATAGTAAGTTATGTTTTGCAGGATGCACGTTTTTTAAGATACTATTTTAGGCATGTTGGAGTACACGATGACAATGTTGGAAAAAATTTTGAGCCAAAGATTAAGGCTTTGATTTA |
| Bundibugyo-5 | 13,426-13,451 | L | GAATGCCACTGTTAGAGGCAGCAGTTTTGTTACCGACCTAGAAAAATACAACTTGGCATTTAGATATGAGTTTACAGCTCCATTTATTGAATACTGTAATCGATGTTATGGTGTAAAAAATTTATTCAATTGGATGCATTATACGATACCGC |
| Bundibugyo-6 | 11,375-11,527 | VP24-L | CTGAGTTGTGGATTACTCCTTTTAAAAGTCTATCAATTTAAGCTTATCACTAATATTAAGGAGGACTTTTTAAATAAGAGCAAGTGTTATGTAGTCTTACTAAGAATGATTTGAGGAAGATTAAGAAAAAGTGCTTGTGGGGTCTTTCCGTTG |
| Bundibugyo-7 | 11,361-11,512 | VP24-L | TCTGGCTAACTAACCTGAGTTGTGGATTACTCCTTTTAAAAGTCTATCAATTTAAGCTTATCACTAATATTAAGGAGGACTTTTTAAATAAGAGCAAGTGTTATGTAGTCTTACTAAGAATGATTTGAGGAAGATTAAGAAAAAGTGCTTGT |
| Bundibugyo-8 | 11,360-11,511 | VP24-L | CTCTGGCTAACTAACCTGAGTTGTGGATTACTCCTTTTAAAAGTCTATCAATTTAAGCTTATCACTAATATTAAGGAGGACTTTTTAAATAAGAGCAAGTGTTATGTAGTCTTACTAAGAATGATTTGAGGAAGATTAAGAAAAAGTGCTTG |
| Bundibugyo-10 | 10,102-10,254 | VP24 | ACTGACTTAATTAGCTTGTAATCAGATAATATCGAAACCAATTTATCATAAGGCATAATTTGTATAAGTGATTTAGGATTTACCCCAGAAGTGAAATAATTCTTAGAATAAAAGACCGACTAGAATATCCTTAAGGCTGTCTAACGTGCCACA |
| Bundibugyo-11 | 4265-4416 | VP35-VP40 | GAGAACGCAAGGCCATATCAGGACCCGATCTCAATTCCAATGCAACCTACTGTTAAGAATAAAATAACCAATGTCCTCTAGCCTTATATGTTCTCAAAAATACAAGTGATGAAGATTAAGAAAAAGCATCCTTTACTTGAGAGGAGCTAATT |
| Bundibugyo-12 | 3879-4030 | VP35 | GATGTAATTCATGCAGAATTTCAGGCCAGCCTTGCTGAAGGAGACTCTCCTCAGTGTGCCCTGATTCAGATAACCAAACGGATTCCTATTTTCCAAGATGCAGCACCACCCGTAATCCATATTCGGTCACGCGGTGATATACCAAAGGCGTG |
| Bundibugyo-14 | 3499-3651 | VP35 | CACTAAATAGATCCTGTGCAGAGATGGTGGCCAAATATGATCTTCTAGTAATGACGACTGGTCGTGCAACTGCCACTGCTGCAGCTACTGAAGCATACTGGGCAGAACATGGACGTCCTCCACCGGGGCCCTCATTGTACGAGGAGGATGCAA |
| Bundibugyo-15 | 3888-4039 | VP35 | CATGCAGAATTTCAGGCCAGCCTTGCTGAAGGAGACTCTCCTCAGTGTGCCCTGATTCAGATAACCAAACGGATTCCTATTTTCCAAGATGCAGCACCACCCGTAATCCATATTCGGTCACGCGGT GATATACCAAAGGCGTGTCAAAAGAG |
| Taï Forest -1 | 17,873-18,024 | L | ATAATCAACAAAGACAAAGTCGAACCCAAACATATCATTTCATTAAAACAATAAAAGGTCGTATTACAAAATTGGTAAATGATTACCTTAAGTTCTTTCTAATAATACAAGCCTTAAAGCACAATTGCACATGGCAAGAGGAACTAAGAGCT |
| Taï Forest -3 | 17,856-18,007 | L | GGAGTTGGTTAATGACTATAATCAACAAAGACAAAGTCGAACCCAAACATATCATTTCATTAAAACAATAAAAGGTCGTATTACAAAATTGGTAAATGATTACCTTAAGTTCTTTCTAATAATACAAGCCTTAAAGCACAATTGCACATGGC |
| Taï Forest -4 | 17,855-18,007 | L | GGGAGTTGGTTAATGACTATAATCAACAAAGACAAAGTCGAACCCAAACATATCATTTCATTAAAACAATAAAAGGTCGTATTACAAAATTGGTAAATGATTACCTTAAGTTCTTTCTAATAATACAAGCCTTAAAGCACAATTGCACATGGC |
| Taï Forest -15 | 3243-3395 | VP35 | ATCTTCAACGACACTGAGCCCCACATAAGCTCAGGGTCCGACTGCCTTCCCAGACCCAAAAACACGGCCCCCCGGACTCGCAACACCCAGACACAGACCGATCCGGTTTGCAATCACAATTTTGAAGACGTTACACAAGCACTAACATCATTA |
| Reston-5 | 18,407-18,558 | L | TGTACTACCAGAGAAATGACCCCAGTTCCTATCTTTAAATAATGATTGTGTGTATTAAATTATTAGTTTATTAGGTTTATGAGTTGGTTACACAGTGAGTATTAGTAATTGAGGATTATGTAGATAGGTAATCTAACACTGAATCACCCATC |
| Reston-6 | 11,333-11,484 | VP24 | AGAACAATGCCTAACTATTGGGCGGCTTCCATTTTTACATGTGTATATAACCAATCTTTTCCTATCTTTGCTTATATTGGTGTAACTTTATTTTAATAACATGTCAATGCTATACTGTTAAGAGAAGGTCTGAGGAAGATTAAGAAAAAGGC |
| Reston-7 | 11,338-11,489 | VP24 | AATGCCTAACTATTGGGCGGCTTCCATTTTTACATGTGTATATAACCAATCTTTTCCTATCTTTGCTTATATTGGTGTAACTTTATTTTAATAACATGTCAATGCTATACTGTTAAGAGAAGGTCTGAGGAAGATTAAGAAAAAGGCCTCGT |
| Sudan-3 | 17,177-17,328 | L | AACACATCGTGGTGAGCTAGAGGTCATATTAAATAACTCAGCTAGTCAAATAACTGATATTACACATCGAGATTGGTTTTCAAATCAAAAAAATAGGATTCCAAATGATGCTGATATTATTACCATGGATGCTGAAACTACAGAAAACTTAG |
| Sudan-4 | 17,290-17,441 | L | ATATTATTACCATGGATGCTGAAACTACAGAAAACTTAGATCGTTCCAGATTATATGAAGCAGTATATACGATTATTTGTAATCATATCAATCCTAAAACTTTGAAAGTGGTCATCTTAAAAGTCTTCCTCAGCGATTTGGATGGGATGTGC |
| Sudan-5 | 17,285-17,436 | L | TGCTGATATTATTACCATGGATGCTGAAACTACAGAAAACTTAGATCGTTCCAGATTATATGAAGCAGTATATACGATTATTTGTAATCATATCAATCCTAAAACTTTGAAAGTGGTCATCTTAAAAGTCTTCCTCAGCGATTTGGATGGGA |
| Sudan-6 | 11,268-11,419 | VP24 | TGTATAGCATGACCCTAGTCATGCCTTTAATTAATACTTAATCTAACAGTTAATATAATGTATAACTTTCCATGTTCAAAGAGTAGTCAAAACAATGTGAGATCCAGTTTCACTCACAGCATCTATTCACTATTTACAGTATGATGAGCCCA |
| Sudan-7 | 9839-9864 | VP24 | GAGAAGGTTCCAAGATTGACTTCAATCCAAACACCTTGCTCTGCCAATTTTCATCTCCTTAAGATATATGATTTTGTTCCTGCGAGATAAGGTTATCAAATAGGGTGTGTATCTCTTTTACATATTTGGGCTCCCACTAGGCTAGGGTTTAT |
| Sudan-8 | 10,355-10,486 | VP24 | CCAAAACGGGAGCTAGAGCAAGGAGTTGTGTTTAGCGACCTATGCAACTTCCTAGTGACTCCAACTGTGCAAGGATGGAAGGTTTACTGGGCTGGACTTGAGTTTGATGTCAACCAAAAGGGTATTACCCTGTTAAATCGTCTTAAAGTGAA |
| Sudan-9 | 10,309-10,460 | VP24 | CCACAGGCCGGTACAACTTGGTAACACCAAAACGGGAGCTAGAGCAAGGAGTTGTGTTTAGCGACCTATGCAACTTCCTAGTGACTCCAACTGTGCAAGGATGGAAGGTTTACTGGGCTGGACTTGAGTTTGATGTCAACCAAAAGGGTATT |
| Sudan-10 | 10,303-10,454 | VP24 | CTAAAGCCACAGGCCGGTACAACTTGGTAACACCAAAACGGGAGCTAGAGCAAGGAGTTGTGTTTAGCGACCTATGCAACTTCCTAGTGACTCCAACTGTGCAAGGATGGAAGGTTTACTGGGCTGGACTTGAGTTTGATGTCAACCAAAAG |
| Sudan-11 | 2971-3124 | VP35 | CCTGATGAATTCTATAGAACTTAGGATTAAGAAAAAATTCATGATGAAGATTAAAACCTTCATCATCCTTTAAAAAGAGAGCTATTCTTTATCTGAATGTCCTTATTAATGTCTAAGAGCTATTATTTTGTACCCTCTTAGCCTAGACACTGCC |
| Sudan-12 | 3503-3655 | VP35 | CAGCTGTGCCGAAATGGTTGCAAAATACGACCTACTGGTGATGACCACTGGGCGAGCAACTGCCACTGCTGCAGCAACAGAAGCATATTGGAATGAACATGGACAAGCACCTCCAGGCCCATCATTGTACGAGGATGATGCTATTAAGGCTAA |
| Sudan-13 | 3468-3493 | VP35 | CAAGACATGGCAAAGACCATATCATCCCTGAATCGCAGCTGTGCCGAAATGGTTGCAAAATACGACCTACTGGTGATGACCACTGGGCGAGCAACTGCCACTGCTGCAGCAACAGAAGCATATTGGAATGAACATGGACAAGCACCTCCAGGC |
| Sudan-14 | 3641-3612 | VP35 | ACCCGTTCAAGACATGGCAAAGACCATATCATCCCTGAATCGCAGCTGTGCCGAAATGGTTGCAAAATACGACCTACTGGTGATGACCACTGGGCGAGCAACTGCCACTGCTGCAGCAACAGAAGCATATTGGAATGAACATGGACAAGCAC |
| Sudan-15 | 3102-3253 | VP35 | ACCCTCTTAGCCTAGACACTGCCCAGCATATAAGCCATGCAGCAGGATAGGACTTATAGACATCATGGACCCGAAGTGTCTGGCTGGTTTTCTGAGCAATTAATGACCGGCAAAATACCGCTAACAGAGGTGTTTGTTGATGTTGAAAACAA |
| Ebola-5 | 12,237-12,388 | L | AGAAGCGGTTCAAGGGCATACACACATTGTTTCTGTTTCTACTGCCGACGTCTTGATAATGTGCAAAGATTTAATTACATGTCGATTCAACACAACTCTAATCTCAAAAATAGCAGAGATTGAGGATCCAGTTTGTTCTGATTATCCCAATT |
| Ebola -6 | 11,121-11,272 | VP24 | TTGAGCTAACTCATATATGCTGACTCAATAGTTATCTTGACATCTCTGCTTTCATAATCAGATATATAAGCATAATAAATAAATACTCATATTTCTTGATAATTTGTTTAACCACAGATAAATCCTCACTGTAAGCCAGCTTCCAAGTTGAC |
| Ebola -7 | 11,117-11,268 | VP24 | CATATTGAGCTAACTCATATATGCTGACTCAATAGTTATCTTGACATCTCTGCTTTCATAATCAGATATATAAGCATAATAAATAAATACTCATATTTCTTGATAATTTGTTTAACCACAGATAAATCCTCACTGTAAGCCAGCTTCCAAGT |
| Ebola -8 | 10,480-10,631 | VP24 | GAGTTTGATGTGACTCACAAAGGAATGGCCCTATTGCATAGACTGAAAACTAATGACTTTGCCCCTGCATGGTCAATGACAAGGAATCTCTTTCCTCATTTATTTCAAAATCCGAATTCCACAATTGAATCACCGCTGTGGGCATTGAGAGT |
| Ebola -9 | 10,073-10,224 | VP24 | TACACATCCCATACATTGTATTAGGGGCAATAATATCTAATTGAACTTAGCCGTTTAAAATTTAGTGCATAAATCTGGGCTAACACCACCAGGTCAACTCCATTGGCTGAAAAGAAGCTTACCTACAACGAACATCACTTTGAGCGCCCTCA |
| Ebola -12 | 3994-4145 | VP35 | ATGCTGCTCCACCTGTCATCCACATCCGCTCTCGAGGTGACATTCCCCGAGCTTGCCAGAAAAGCTTGCGTCCAGTCCCACCATCGCCCAAGATTGATCGAGGTTGGGTATGTGTTTTTCAGCTTCAAGATGGTAAAACACTTGGACTCAAA |
| Ebola -13 | 3991-4142 | VP35 | AAGATGCTGCTCCACCTGTCATCCACATCCGCTCTCGAGGTGACATTCCCCGAGCTTGCCAGAAAAGCTTGCGTCCAGTCCCACCATCGCCCAAGATTGATCGAGGTTGGGTATGTGTTTTTCAGCTTCAAGATGGTAAAACACTTGGACTC |
| Ebola -14 | 3985-4136 | VP35 | TCTTCCAAGATGCTGCTCCACCTGTCATCCACATCCGCTCTCGAGGTGACATTCCCCGAGCTTGCCAGAAAAGCTTGCGTCCAGTCCCACCATCGCCCAAGATTGATCGAGGTTGGGTATGTGTTTTTCAGCTTCAAGATGGTAAAACACTT |
| Musoke-1 | 18,139-18,290 | L | ACAATTCACCTCTACTAGACTTGAGAAATCACTTTATTTGCTCATTAAGGGGAAAGATAACTAAATATTACAATGATATATTAAAGTTAAATCTAGTCATCAAGGCAGTAGAAAAAGGTAAAAATTGGTCACAACTTGTTGAGATCCTTCCT |
| Musoke-4 | 17,480-17,631 | L | TGCTTTGCTTGAATCAAGGAGACTAATATTGAACAACCTAACTATCCAAATTACAGATATTACAAATCCATTATGGCTAGATTCTGTAATACAATATTTACCTGAAGATAGTGACATTCTTACAATGGACGCAGAGACCACCAAGGATGAAA |
| Musoke-6 | 2228-2379 | NP | TTGATATGACTCATCCTCAGATCACAGCAATCAAATTTATTTGAATATTTGAACCACCTTTTAGTATCCTATTACTTGTTACTATTGTGTGAGACAACATAAGCCATCAAATAACAATCACGGGCAAGGACTGGGCATACTATGGTGGTCTT |
| Musoke-7 | 2223-2374 | NP | CTATTTTGATATGACTCATCCTCAGATCACAGCAATCAAATTTATTTGAATATTTGAACCACCTTTTAGTATCCTATTACTTGTTACTATTGTGTGAGACAACATAAGCCATCAAATAACAATCACGGGCAAGGACTGGGCATACTATGGTG |
| Musoke-8 | 941-1094 | NP | CGGGTTCTGAATTTATCAGGGATTAACAACCTCGAACATGGACTCTATCCTCAGCTTTCAGCAATTGCGCTGGGTGTGGCAACAGCACACGGCAGTACATTGGCTGGTGTCAATGTTGGCGAACAATATCAACAACTACGAGAGGCGGCACATG |
| Musoke-10 | 837-988 | NP | CACTACATCCTTTGGTGCGGACCTCCAAAGTAAAAAATGAAGTTGCTAGTTTCAAGCAGGCGTTGAGCAACCTAGCCCGACATGGGGAATACGCACCATTTGCACGGGTTCTGAATTTATCAGGGATTAACAACCTCGAACATGGACTCTAT |
| Musoke-11 | 4312-4465 | VP35-VP40 | GACCTCAAGACGCTTATTCATAGTATATTATATGATTTTTTATAAGTTTAAGATATCTTAAATTATACCCACAAAAGATACTGTTTTAATTAAGAAAAACTATGAAGAACATTAAGAAGATCTTTCTTTCGTAGTGTTCTTTTACTGGAAGGAG |
| Musoke-12 | 4312-4464 | VP35-VP40 | GACCTCAAGACGCTTATTCATAGTATATTATATGATTTTTTATAAGTTTAAGATATCTTAAATTATACCCACAAAAGATACTGTTTTAATTAAGAAAAACTATGAAGAACATTAAGAAGATCTTTCTTTCGTAGTGTTCTTTTACTGGAAGGA |
| Musoke-14 | 3953-4104 | VP35 | GCAAGGGAAGTGATCTTTCCGAGTTGATACAAAGACACTAAACATTTCAAAAGCATGTATGTGGACAAAACATAATTAGACCATCTTAATTGGAGTAGTAATTTATTTCTGTCTTAAATGTGATTTTCACTTTAAAAGCGTTAAATGGTGAT |
| Musoke-15 | 3504-3655 | VP35 | ATGCAGCCGACAAGATGTCGAAGGTTCTTGAACTCAGTGAGGAAACGTTCTCCAAGCCAAACCTTTCAGCTAAGGATTTAGCCCTTTTATTGTTTACCCATCTACCCGGCAACAACACTCCATTCCATATCCTAGCTCAGGTCCTTTCAAAA |
| Angola-4 | 17,480-17,631 | L | TGTTTTGCTTGAATCAAGAAGACTAATATTGAACAATCTAACTATCCAAATTACAGATATTACAAATCCACTATGGCTAGATTCTGTAATACAATATTTACCTGAAGATAGTGACATTCTTACAATGGATGCAGAGACCACTAAGGATGAAA |
| Angola-5 | 17,480-17,627 | L | TAAGTGTTTTGCTTGAATCAAGAAGACTAATATTGAACAATCTAACTATCCAAATTACAGATATTACAAATCCACTATGGCTAGATTCTGTAATACAATATTTACCTGAAGATAGTGACATTCTTACAATGGATGCAGAGACCACTAAGGAT |
| Angola-8 | 941-1069 | NP | CGGGTTTTGAATTTATCAGGGATTAACAATCTCGAACATGGACTCTATCCTCAGCTCTCAGCAATTGCGCTGGGTGTAGCGACAGCACATGGCAGTACATTGGCTGGTGTCAATGTCGGCGAACAATATCAACAGCTACGAGAGGCAGCACATG |
| Angola-9 | 941-1092 | NP | CGGGTTTTGAATTTATCAGGGATTAACAATCTCGAACATGGACTCTATCCTCAGCTCTCAGCAATTGCGCTGGGTGTAGCGACAGCACATGGCAGTACATTGGCTGGTGTCAATGTCGGCGAACAATATCAACAGCTACGAGAGGCAGCACA |
| Angola-11 | 4312-4465 | VP35-VP40 | TAATTCAATACGTTTAGTCATAGTATATTGTAGGATTTTTTATAAGTTTAGAATACCTTAAATCATACCCACAAAAAATACTGTTTTAATTAAGAAAAACTATGAAGAACATTAAGAAGATCTTTCTCTCGTAGTGTTCTTTTACTGGAAGAAG |
| Angola-12 | 4312-4464 | VP35-VP40 | TAATTCAATACGTTTAGTCATAGTATATTGTAGGATTTTTTATAAGTTTAGAATACCTTAAATCATACCCACAAAAAATACTGTTTTAATTAAGAAAAACTATGAAGAACATTAAGAAGATCTTTCTCTCGTAGTGTTCTTTTACTGGAAGAA |
| Angola-14 | 3953-4079 | VP35 | GCAAGGGAAGTTATCTTCCCGAGTTGATACAAAGACACTAAACATTTCAAAAGCATGTATGTGGGCAAAACGTAACTAGACCATCTTGATTGAAGTAGTAATTTATTTCTGTCTTAAATGTGATTTTCACTTCAAAAGAGTTAAATGGTGAT |
| Angola-15 | 3504-3655 | VP35 | ATGCAGCCGACAAGATGTCGAAGGTCCTTGAACTCAGTGAGGAAACGTTCTCCAAGCCAAACCTCTCAGCCAAGGATTTAGCCCTTTTATTGTTTACCCATCTACCCGGCAACAACACTCCATTCCACATCCTAGCTCAGGTCCTTTCAAAA |
| Ci67-3 | 18,168-18,319 | L | CTTTATTTGCTCATTGAGGGGAAAGATAACCAAGTATTACAATGATATATTAAAGTTAAATCTAGTTATCAAGGCAGTAGAGAAAGGTAAAAATTGGTCACAACTTGTTGAGACCCTTCCTAATATGCATTCAGTCTGCATAGTACACGTGG |
| Ci67-5 | 17,474-17,625 | L | TAAATGCTTTGCTTGAATCAAGAAGATTAATATTGAACAACCTAACTATCCAAATTACAGATATTACAAGTCCACTATGGCTAGATTCTGTAATACAATACTTACCTGAAGATAGCGACATTCTTACAATGGACGCAGAGACCACTAAAGAT |
| Ci67-6 | 2227-2375 | NP | TTGATCCGATTCGTCCTCAGATCACAGTAATCAAATTTATTTGAATATTCAAACTACTCTTTAGGATCCCATCACTTGTTACTATTGTGTGAGACAACACAAGCCATCAAATAACAATCACGGGCAAGAACCGGGCACACCATGGTGAT |
| Ci67-7 | 2222-2373 | NP | CTACTTTGATCCGATTCGTCCTCAGATCACAGTAATCAAATTTATTTGAATATTCAAACTACTCTTTAGGATCCCATCACTTGTTACTATTGTGTGAGACAACACAAGCCATCAAATAACAATCACGGGCAAGAACCGGGCACACCATGGTG |
| Ci67-8 | 940-1093 | NP | CGGGTTCTGAATTTATCAGGGATTAACAACCTCGAACATGGACTCTATCCTCAGCTTTCAGCGATTGCGCTGGGTGTTGCAACAGCACACGGCAGTACATTGGCTGGTGTCAATGTTGGCGAACAGTATCAACAGCTACGAGAGGCGGCACATG |
| Ci67-9 | 940-1091 | NP | CGGGTTCTGAATTTATCAGGGATTAACAACCTCGAACATGGACTCTATCCTCAGCTTTCAGCGATTGCGCTGGGTGTTGCAACAGCACACGGCAGTACATTGGCTGGTGTCAATGTTGGCGAACAGTATCAACAGCTACGAGAGGCGGCACA |
| Ci67-14 | 3952-4103 | VP35 | GCAGGGGGAGTGATCTTTCCGAGTTGATACAAAGACACTAAACATTTCAAAAGCATATATGTGGGCAAAACGTGACTAGACCATCTTAATAGAAGTAGTAATTTATTTCTGTCTTAAGTGTGATTTTCACCTTGAAAGAGTTAAATGGTGAT |
| Ci67-15 | 3503-3654 | VP35 | ATGCAGCCGACAAGATGTCGAAAGTTCTTGAACTCAGTGAGGAGACGTTCTCCAAGCCAAATCTTTCAGCTAAGGATTTAGCCCTTTTGTTGTTTACCCATCTACCCGGCAACAACACTCCATTCCATATCCTAGCTCAAGTCCTTTCGAAA |

^1^Underlined sequence is the probe arm hybridization site
