# Supplementary material for: Development and Evaluation of a Panel of Filovirus Sequence Capture Probes for Pathogen Detection by Next-Generation Sequencing
Source: PLoS One. 2014 Sep 10;9(9):e107007. doi: 10.1371/journal.pone.0107007 (PMC4160210; doi:10.1371/journal.pone.0107007)
Supplement: Table S2 — Detailed read mapping for the nonhuman primate clinical sera samples using the filovirus probe panel. (DOCX) [file pone.0107007.s003.docx]

**Table S2. Detailed read mapping for the nonhuman primate clinical sera samples using the filovirus probe panel.**

| **NHP** | **Day post challenge** | **reads** | **Ebola** | | **Sudan** | | **Taï Forest** | | **Bundibugyo** | | **Reston** | | **Musoke** | | **Angola** | | **Ci67** | |
| --- | --- | --- | --- | --- | --- | --- | --- | --- | --- | --- | --- | --- | --- | --- | --- | --- | --- | --- |
|  |  |  | **mapped** | **%** | **mapped** | **%** | **mapped** | **%** | **mapped** | **%** | **mapped** | **%** | **mapped** | **%** | **mapped** | **%** | **mapped** | **%** |
| NHP#1 | -6 | 307,516 | 186 | 0.06 | 0 | 0 | 0 | 0 | 0 | 0 | 0 | 0 | 0 | 0 | 0 | 0 | 0 | 0 |
|  | 0 | 176,281 | 156 | 0.09 | 0 | 0 | 0 | 0 | 0 | 0 | 0 | 0 | 0 | 0 | 0 | 0 | 0 | 0 |
|  | 3 | 244,579 | 58 | 0.02 | 0 | 0 | 0 | 0 | 0 | 0 | 0 | 0 | 0 | 0 | 0 | 0 | 0 | 0 |
|  | 6 | 606,415 | 19 | 0.003 | 0 | 0 | 0 | 0 | 0 | 0 | 0 | 0 | 0 | 0 | 0 | 0 | 0 | 0 |
|  | 10 | 708,758 | 186 | 0.03 | 0 | 0 | 0 | 0 | 0 | 0 | 0 | 0 | 0 | 0 | 0 | 0 | 0 | 0 |
|  | 14 | 408,602 | 964 | 0.24 | 0 | 0 | 0 | 0 | 0 | 0 | 0 | 0 | 0 | 0 | 0 | 0 | 0 | 0 |
|  | 17 | 111,689 | 380 | 0.34 | 0 | 0 | 0 | 0 | 0 | 0 | 0 | 0 | 0 | 0 | 0 | 0 | 0 | 0 |
| NHP#2 | -6 | 388,936 | 56 | 0.01 | 0 | 0 | 0 | 0 | 1 | 0.00 | 0 | 0 | 0 | 0 | 0 | 0 | 0 | 0 |
|  | 0 | 311,484 | 1,060 | 0.34 | 0 | 0 | 0 | 0 | 0 | 0 | 0 | 0 | 0 | 0 | 0 | 0 | 0 | 0 |
|  | 3 | 655,910 | 143 | 0.02 | 0 | 0 | 0 | 0 | 0 | 0 | 0 | 0 | 0 | 0 | 0 | 0 | 0 | 0 |
|  | 6 | 376,673 | 179 | 0.05 | 0 | 0 | 0 | 0 | 0 | 0 | 0 | 0 | 0 | 0 | 0 | 0 | 0 | 0 |
|  | 10 | 210,470 | 161,342 | 76.66 | 0 | 0 | 0 | 0 | 0 | 0 | 0 | 0 | 0 | 0 | 0 | 0 | 0 | 0 |
| NHP#3 | -6 | 253,187 | 5,906 | 2.33 | 0 | 0 | 0 | 0 | 0 | 0 | 0 | 0 | 0 | 0 | 0 | 0 | 0 | 0 |
|  | 0 | 41,293 | 277 | 0.67 | 0 | 0 | 0 | 0 | 0 | 0 | 0 | 0 | 0 | 0 | 0 | 0 | 0 | 0 |
|  | 3 | 167,939 | 79 | 0.05 | 0 | 0 | 0 | 0 | 0 | 0 | 0 | 0 | 0 | 0 | 0 | 0 | 0 | 0 |
|  | 6 | 576,576 | 382,904 | 66.41 | 0 | 0 | 0 | 0 | 0 | 0 | 0 | 0 | 0 | 0 | 0 | 0 | 0 | 0 |
| NHP#4 | -6 | 271,068 | 192 | 0.07 | 0 | 0 | 0 | 0 | 0 | 0 | 0 | 0 | 0 | 0 | 0 | 0 | 0 | 0 |
|  | 0 | 218,743 | 720 | 0.33 | 0 | 0 | 0 | 0 | 0 | 0 | 0 | 0 | 0 | 0 | 0 | 0 | 0 | 0 |
|  | 3 | 424,157 | 343 | 0.08 | 0 | 0 | 0 | 0 | 0 | 0 | 0 | 0 | 0 | 0 | 0 | 0 | 0 | 0 |
|  | 6 | 627,136 | 273,055 | 43.54 | 0 | 0 | 0 | 0 | 0 | 0 | 0 | 0 | 0 | 0 | 0 | 0 | 0 | 0 |
|  | 7 | 768,083 | 673,125 | 87.64 | 0 | 0 | 1 | 0.00 | 0 | 0 | 0 | 0 | 0 | 0 | 0 | 0 | 0 | 0 |
| NHP#5 | -6 | 405,038 | 178 | 0.04 | 0 | 0 | 0 | 0 | 0 | 0 | 0 | 0 | 0 | 0 | 0 | 0 | 0 | 0 |
|  | 0 | 201,972 | 28 | 0.01 | 0 | 0 | 0 | 0 | 0 | 0 | 0 | 0 | 0 | 0 | 0 | 0 | 0 | 0 |
|  | 3 | 370,957 | 74 | 0.02 | 0 | 0 | 0 | 0 | 0 | 0 | 0 | 0 | 0 | 0 | 0 | 0 | 0 | 0 |
|  | 6 | 399,371 | 344 | 0.09 | 0 | 0 | 0 | 0 | 0 | 0 | 0 | 0 | 0 | 0 | 0 | 0 | 0 | 0 |
|  | 9 | 321,404 | 27 | 0.01 | 0 | 0 | 0 | 0 | 0 | 0 | 0 | 0 | 0 | 0 | 0 | 0 | 0 | 0 |
| NHP#6 | -6 | 153,441 | 39 | 0.03 | 0 | 0 | 0 | 0 | 0 | 0 | 0 | 0 | 0 | 0 | 0 | 0 | 0 | 0 |
|  | 0 | 431,312 | 83 | 0.02 | 0 | 0 | 0 | 0 | 0 | 0 | 0 | 0 | 0 | 0 | 0 | 0 | 0 | 0 |
|  | 3 | 202,745 | 50 | 0.02 | 0 | 0 | 0 | 0 | 0 | 0 | 0 | 0 | 0 | 0 | 0 | 0 | 0 | 0 |
|  | 6 | 388,065 | 117 | 0.03 | 0 | 0 | 0 | 0 | 0 | 0 | 0 | 0 | 0 | 0 | 0 | 0 | 0 | 0 |
|  | 10 | 144,059 | 95 | 0.07 | 0 | 0 | 0 | 0 | 0 | 0 | 0 | 0 | 0 | 0 | 0 | 0 | 0 | 0 |
| PTC1 | | 867,784 | 620,431 | 71.50 | 0 | 0 | 0 | 0 | 2 | 0.00 | 0 | 0 | 0 | 0 | 0 | 0 | 0 | 0 |
| PTC2 | | 567,493 | 400,541 | 70.58 | 0 | 0 | 0 | 0 | 3 | 0.00 | 1 | 0.00 | 0 | 0 | 0 | 0 | 0 | 0 |
| NTC1 | | 260,867 | 91 | 0.03 | 0 | 0 | 0 | 0 | 0 | 0 | 0 | 0 | 0 | 0 | 0 | 0 | 0 | 0 |
| NTC2 | | 167,835 | 64 | 0.04 | 0 | 0 | 0 | 0 | 0 | 0 | 0 | 0 | 0 | 0 | 0 | 0 | 0 | 0 |
