# Supplementary material for: Development and Evaluation of a Panel of Filovirus Sequence Capture Probes for Pathogen Detection by Next-Generation Sequencing
Source: PLoS One. 2014 Sep 10;9(9):e107007. doi: 10.1371/journal.pone.0107007 (PMC4160210; doi:10.1371/journal.pone.0107007)
Supplement: Table S3 — Detailed read mapping for the human clinical sera samples using the filovirus probe panel (run 1). (DOCX) [file pone.0107007.s004.docx]

**Table S3. Detailed read mapping for the human clinical sera samples using the filovirus probe panel (run 1).**

| **RNA** | **matrix** | **reads** | **Ebola** | | **Sudan** | | **Taï Forest** | | **Bundibugyo** | | **Reston** | | **Musoke** | | **Angola** | | **Ci67** | |
| --- | --- | --- | --- | --- | --- | --- | --- | --- | --- | --- | --- | --- | --- | --- | --- | --- | --- | --- |
|  |  |  | **mapped** | **%** | **mapped** | **%** | **mapped** | **%** | **mapped** | **%** | **mapped** | **%** | **mapped** | **%** | **mapped** | **%** | **mapped** | **%** |
| 2012-1 | serum | 145,553 | 0 | 0 | 0 | 0 | 0 | 0 | 845 | 0.581 | 0 | 0 | 0 | 0 | 0 | 0 | 0 | 0 |
|  | supernatant-1 | 638,437 | 1 | 0.00 | 0 | 0 |  | 0 | 568,626 | 89.065 | 0 | 0 | 0 | 0 | 0 | 0 | 0 | 0 |
|  | supernatant-2 | 840,456 | 2 | 0.00 | 0 | 0 | 6 | 0.00 | 569,439 | 67.754 | 0 | 0 | 0 | 0 | 0 | 0 | 0 | 0 |
| 2012-16 | serum | 828,309 | 0 | 0 | 0 | 0 | 0 | 0 | 1,997 | 0.241 | 0 | 0 | 0 | 0 | 0 | 0 | 0 | 0 |
| 2012-91 | serum | 255,862 | 0 | 0 | 0 | 0 | 0 | 0 | 431 | 0.168 | 0 | 0 | 0 | 0 | 0 | 0 | 0 | 0 |
| 2012-95 | serum | 248,909 | 0 | 0 | 0 | 0 | 1 | 0.00 | 811 | 0.326 | 0 | 0 | 0 | 0 | 0 | 0 | 0 | 0 |
| 2012-99 | serum | 201,613 | 0 | 0 | 0 | 0 | 1 | 0.00 | 1,223 | 0.607 | 0 | 0 | 0 | 0 | 0 | 0 | 0 | 0 |
| 2012-120 | serum | 211,015 | 0 | 0 | 0 | 0 | 0 | 0 | 1,309 | 0.620 | 0 | 0 | 0 | 0 | 0 | 0 | 0 | 0 |
|  | supernatant | 604,371 | 2 | 0.00 | 2 | 0.00 | 1 | 0.00 | 527,607 | 87.299 | 0 | 0 | 0 | 0 | 0 | 0 | 0 | 0 |
| 2012-147 | serum | 183,639 | 10 | 0.00 | 0 | 0 | 0 | 0 | 2,070 | 1.127 | 0 | 0 | 0 | 0 | 0 | 0 | 0 | 0 |
|  | supernatant | 967,611 | 5 | 0.00 | 0 | 0 | 9 | 0 | 852,653 | 88.119 | 0 | 0 | 0 | 0 | 0 | 0 | 0 | 0 |
| 2012-153 | serum | 177,170 | 0 | 0 | 0 | 0 | 0 | 0 | 1,303 | 0.735 | 0 | 0 | 0 | 0 | 0 | 0 | 0 | 0 |
|  | supernatant | 709,431 | 2 | 0.00 | 0 | 0 | 7 | 0.00 | 565,642 | 79.732 | 0 | 0 | 0 | 0 | 0 | 0 | 0 | 0 |
| 2012-176 | serum | 324,518 | 0 | 0 | 0 | 0 | 2 | 0.00 | 842 | 0.259 | 0 | 0 | 0 | 0 | 0 | 0 | 0 | 0 |
| 2012-198 | serum | 321,721 | 0 | 0 | 0 | 0 | 1 | 0.00 | 1,182 | 0.367 | 0 | 0 | 0 | 0 | 0 | 0 | 0 | 0 |
|  | supernatant | 802,109 | 2 | 0.00 | 0 | 0 | 7 | 0.00 | 713,666 | 88.974 | 0 | 0 | 0 | 0 | 0 | 0 | 0 | 0 |
| PTC | | 727,421 | 1 | 0.00 | 1 | 0.00 | 10 | 0.00 | 684,988 | 94.167 | 0 | 0.00 | 1 | 0.00 | 0 | 0 | 1 | 0.00 |
| NTC1 | | 153,857 | 0 | 0 | 0 | 0 | 0 | 0 | 934 | 0.607 | 0 | 0 | 0 | 0 | 0 | 0 | 0 | 0 |
| NTC2 | | 170,488 | 0 | 0 | 0 | 0 | 0 | 0 | 572 | 0.336 | 0 | 0 | 0 | 0 | 0 | 0 | 0 | 0 |
| NTC3 | | 311,773 | 0 | 0 | 0 | 0 | 0 | 0 | 324 | 0.104 | 0 | 0 | 0 | 0 | 0 | 0 | 0 | 0 |
| NTC4 | | 307,239 | 0 | 0 | 0 | 0 | 1 | 0.00 | 806 | 0.262 | 0 | 0 | 0 | 0 | 0 | 0 | 0 | 0 |
| NTC5 | | 133,303 | 0 | 0 | 0 | 0 | 1 | 0.00 | 1,462 | 1.097 | 0 | 0 | 0 | 0 | 0 | 0 | 0 | 0 |
|  |  |  |  |  |  |  | **Average** | | 819.6 | 0.481 |  |  |  |  |  |  |  |  |
|  |  |  |  |  |  |  | **STDEV** | | 428.04 | 0.39 |  |  |  |  |  |  |  |  |
|  |  |  |  |  |  |  | **Cutoff** | | 2103.722 | 1.649 |  |  |  |  |  |  |  |  |
