# Supplementary material for: Development and Evaluation of a Panel of Filovirus Sequence Capture Probes for Pathogen Detection by Next-Generation Sequencing
Source: PLoS One. 2014 Sep 10;9(9):e107007. doi: 10.1371/journal.pone.0107007 (PMC4160210; doi:10.1371/journal.pone.0107007)
Supplement: Table S4 — Detailed read mapping for the human clinical sera samples using the filovirus probe panel (run 2). (DOCX) [file pone.0107007.s005.docx]

**Table S4. Detailed read mapping for the human clinical sera samples using the filovirus probe panel (run 2).**

| **RNA** | **matrix** | **reads** | **Ebola** | | **Sudan** | | **Taï Forest** | | **Bundibugyo** | | **Reston** | | **Musoke** | | **Angola** | | **Ci67** | |
| --- | --- | --- | --- | --- | --- | --- | --- | --- | --- | --- | --- | --- | --- | --- | --- | --- | --- | --- |
|  |  |  | **mapped** | **%** | **mapped** | **%** | **mapped** | **%** | **mapped** | **%** | **mapped** | **%** | **mapped** | **%** | **mapped** | **%** | **mapped** | **%** |
| 2012-1 | serum | 208,322 | 0 | 0 | 0 | 0 | 0 | 0 | 320 | 0.154 | 0 | 0 | 0 | 0 | 0 | 0 | 0 | 0 |
| 2012-16 | serum | 1,132,782 | 0 | 0 | 0 | 0 | 0 | 0 | 825 | 0.073 | 0 | 0 | 0 | 0 | 0 | 0 | 0 | 0 |
| 2012-91 | serum | 344,943 | 0 | 0 | 0 | 0 | 0 | 0 | 283 | 0.082 | 0 | 0 | 0 | 0 | 0 | 0 | 0 | 0 |
| 2012-95 | serum | 343,551 | 0 | 0 | 0 | 0 | 0 | 0 | 364 | 0.106 | 0 | 0 | 0 | 0 | 0 | 0 | 0 | 0 |
| 2012-99 | serum | 280,256 | 0 | 0 | 0 | 0 | 0 | 0 | 965 | 0.344 | 0 | 0 | 0 | 0 | 0 | 0 | 0 | 0 |
| 2012-120 | serum | 279,390 | 0 | 0 | 0 | 0 | 0 | 0 | 800 | 0.286 | 0 | 0 | 0 | 0 | 0 | 0 | 0 | 0 |
| 2012-147 | serum | 243,107 | 0 | 0 | 0 | 0 | 0 | 0 | 870 | 0.358 | 0 | 0 | 0 | 0 | 0 | 0 | 0 | 0 |
| 2012-153 | serum | 227,555 | 0 | 0 | 0 | 0 | 0 | 0 | 947 | 0.416 | 0 | 0 | 0 | 0 | 0 | 0 | 0 | 0 |
| 2012-176 | serum | 447,698 | 0 | 0 | 0 | 0 | 0 | 0 | 294 | 0.066 | 0 | 0 | 0 | 0 | 0 | 0 | 0 | 0 |
| 2012-198 | serum | 447,698 | 0 | 0 | 0 | 0 | 0 | 0 | 676 | 0.151 | 0 | 0 | 0 | 0 | 0 | 0 | 0 | 0 |
| PTC | | 119,617 | 0 | 0 | 0 | 0 | 0 | 0 | 102,060 | 85.322 | 0 | 0 | 0 | 0 | 0 | 0 | 0 | 0 |
| NTC1 | | 292,952 | 0 | 0 | 0 | 0 | 0 | 0 | 369 | 0.126 | 0 | 0 | 0 | 0 | 0 | 0 | 0 | 0 |
| NTC2 | | 232,024 | 0 | 0 | 0 | 0 | 0 | 0 | 16 | 0.007 | 0 | 0 | 0 | 0 | 0 | 0 | 0 | 0 |
| NTC3 | | 430,436 | 0 | 0 | 0 | 0 | 0 | 0 | 13 | 0.003 | 0 | 0 | 0 | 0 | 0 | 0 | 0 | 0 |
| NTC4 | | 412,609 | 0 | 0 | 0 | 0 | 0 | 0 | 40 | 0.010 | 0 | 0 | 0 | 0 | 0 | 0 | 0 | 0 |
| NTC5 | | 148,100 | 0 | 0 | 0 | 0 | 0 | 0 | 21 | 0.014 | 0 | 0 | 0 | 0 | 0 | 0 | 0 | 0 |
|  |  |  |  |  |  |  | **Average** | | 92 | .0.3 |  |  |  |  |  |  |  |  |
|  |  |  |  |  |  |  | **STDEV** | | 155.31 | .0.5 |  |  |  |  |  |  |  |  |
|  |  |  |  |  |  |  | **Cutoff** | | 557.74 | 0.19 |  |  |  |  |  |  |  |  |
